# Supplementary material for: The global flood protection savings provided by coral reefs
Source: Nat Commun. 2018 Jun 12;9:2186. doi: 10.1038/s41467-018-04568-z (PMC5997709; doi:10.1038/s41467-018-04568-z)
Supplement: Supplementary file 1 — Supplementary Information [file 41467_2018_4568_MOESM1_ESM.pdf]

Supporting Information
for
The Global Flood Protection Savings Provided by Coral Reefs

Michael W. Beck, Iñigo J. Losada, Pelayo Menéndez, Borja G. Reguero, Pedro Díaz-Simal, Felipe Fernández

Supplementary Methods

Overview. To estimate the role of coral reefs in coastal protection, we built on the methods from prior work that examines the effects of storm flooding on people and built capital across large regions[1]. The main steps are summarized here and then we describe key aspects of the models including the sensitivity analyses.

Define coastal profiles and study units. We delineated cross-shore profiles every 2 km for all coral reefs globally, and grouped these into 20 km study units across >71,000km of coastline with coral reefs (see Supplementary Figure 3).

Estimate offshore hydrodynamics. We identified sea states offshore for each profile from the combined effects of waves, astronomical tides, storm surge and mean sea level. We used global wave and sea level numerical hindcast datasets that cover the period from 1979 to 2010, which have been validated with instrumental data[2-5].

Estimate nearshore hydrodynamics and the effects of reefs. At each profile, we propagated the waves through the reef profiles, using a propagation model that accounts for shoaling, breaking and the friction induced by the coral reefs. From the wave propagation, we calculate the wave run-up on the shore[6-10]. Reef bathymetry was estimated by the SeaWiFS project[11,12].

Define extreme water levels along the shore. We combined run-up and sea level (storm surge, tides and mean sea level) to estimate flood heights at the coastline[13] and fit a general extreme value distribution[14,15]. We then calculated the flood heights that correspond with the following storm return periods: 10-, 25-, 50- and 100-year.

Identify land, people and built capital flooded. At each profile and for each flood height return period, we calculated the flooding levels on land by intersecting the flood height with a global digital elevation model[16] starting with a bathtub approach and ensuring hydraulic connectivity between flood points at a 90m resolution. We then developed a flood envelope across each 20 km study and calculated the land, people[17] and built capital within this envelope[1,18].

Develop flooding scenario with and without reefs. We repeated the steps above for two reef bathymetry scenarios: "with reef" under current conditions and with reef bathymetric profile reduced by 1m ("without reef"). We also estimated friction under current conditions and reduced this factor for a less rugose coral surface[6].

Identify relative effects of climate change on flooding. We also considered the effects of reef loss relative to climate change by comparing the land flooded globally with a third scenario that considers 1 m reef loss and sea level rise[19] corresponding to the high emissions scenario (Representative Concentration Pathway, RCP 8.5) for different storm return period.

Coastal profiles and study units. We divided the coral reef coastlines into four regions (see Supplementary Figure 4). These regions were further subdivided into 39 sub-regions considering dominant coastal typologies in the area (e.g., continental or small islands coasts) and for analysis and computation purposes.

To identify coastlines, we used the NOAA Global Self-consistent, Hierarchical, High-resolution Geography Database (GSHHG), which provides 5 different resolutions of the global coastline,

ranging from 0.1 to a coarse 25 km resolution. We chose different coastline resolutions on a case-by-case basis for the 39 sub-regions, depending on coastal sinuosity, representativeness of the coastal transects and computational efficiency. The intermediate resolution (1 km) was used in most of the sub-regions.

We divided the coastline in to 2-km cross-shore profiles globally. These profiles were defined orthogonal from the shore to deep water (e.g., see Supplementary Figure 5). As the initial points of all profiles had to be in deep water, the length of the profiles varied. We aggregated the 2-km coastal profiles into larger "study units" that were approximately 20 km wide (along the shoreline) for analysis and representation purposes. The study units were identified by: (1) a coastline segment 20 km wide, (2) a landward buffer parallel to the coast at variable distances (ranging from 1 to 20 km) and (3) a seaward buffer (parallel to the coast) at variable distances (from 30 to 100 km). Irregularities in the coastline such as estuaries and bays resulted in study units that were sometimes wider than 20 km. Supplementary Figure 6 shows examples of these study units in the Caribbean.

Offshore hydrodynamics. Hydrodynamic conditions at offshore locations (Supplementary Figure 3) are obtained by combining global data on wave parameters, mean sea level (MSL), astronomic tide (AT), and storm surge (SS) in to 280,320 1-hourly sea states covering the period 1979-2010. Each single dataset has been used previously in global climatology studies and validated with buoy measurements, satellite altimetry and tidal gauges [2,3]. To propagate the offshore data to the nearshore and calculate flooding, the 32-year long time series of offshore wave and sea level data are classified into a set of 500 sea state conditions using prior published, data-mining based methods[13,20].

Component	Database	Variables	Spatial resolution	Temporal coverage	Temporal resolution	Reference
Waves	GOW1.0	Hs (significant wave height), Tm (mean period), Tp (peak period), Dir (wave direction)	1^ox1^o	1948-2010	Hourly	[21]
Mean Sea Level (MSL)		MSL	1°	1950-2010	Monthly	[22,23]
Storm surge (SS)	DAC	Maximum SS	2°	1871-2010	Daily	[24], [25]
Astronomical Tide (AT)	GOT	AT	0.25^o	no limit	Hourly	Harmonic analysis based on (TPX07.2) Topex/Poseidon and tidal gauges[2]

Nearshore hydrodynamics data. We used the global ETOPO bathymetry[26] and combined it with the SeaWiFS (Sea-Viewing Wide Field-of-View Sensor) bathymetry for coral reefs[11,12]. SeaWiFS reef bathymetry data has a 1 km spatial resolution and was collected over the period 1997-2002. The current SeaWiFS-derived depth map, updated on 6 November 2002, represents the contribution of 28,937 Global Area Coverage (GAC) resolution files. Each SeaWiFS map pixel (0.01 by 0.01 degrees) averages data from no fewer than four input pixels, and many had more than 200 input pixels.

Nearshore hydrodynamics reef wave model. The reef model assumes a longshore uniform, two-dimensional vertical (2DV) coordinate system, with waves propagating shoreward. Wave propagation over the reef is calculated from linear wave theory. Wave propagation is modeled at shore-perpendicular one-dimensional transects, therefore along shore processes such as longshore currents are neglected. The evolution of a wavefield of root-mean square (rms) wave height H with weak mean currents is computed by solving the wave energy balance equation:

$$\partial E_w C_g / \partial x = -(D_b + D_f + D_v) \quad (1)$$

where E_w is the wave energy density and C_g the group velocity. The dissipation of wave energy flux is caused by wave breaking (D_b), bottom friction (D_f), and the presence of vegetation in the water column (D_v), which is not considered in this study. Equation (1) is widely applied in coastal studies to assess wave propagation (e.g. SWAN)[27] and previously applied to reef environments [28]. As applied here, D_b and D_f are expressed following Thornton and Guza [29]:

$$D_b = \frac{3\sqrt{\pi}}{16} \rho g \frac{B^3 \cdot f_p}{\gamma^4 \cdot h^5} \cdot H^7 \quad (2)$$

$$D_f = \frac{f_w}{16\sqrt{\pi}} \left(\frac{\sigma}{\sinh(\mathbf{k}h)} \right)^3 H^3 \quad (3)$$

where ρ is water density, g the constant of gravity, $\mathbf{k}$ the wave number, σ the angular wave frequency and f_p the peak frequency. The breaking coefficient B and breaker index γ have the default values of 1.0 and 0.78 and the bottom friction coefficient f_w is taken as 0.01 for sand beds [8,29]. In our model, we implement recent studies on wave transformation by coral reefs[9,30] and replace the breaker index (γ) by an expression where h/H provides the relationship between water depth and wave height at breaking conditions:

$$\gamma_{coral} = 0.23 tanh \left[2.3143 \left(1.4 - \frac{h}{H} \right) + 3.6522 \right] \quad 0 < \frac{h}{H} < 2.8 \quad (4)$$

As the incident waves propagate into shallow water on the reef flat, they also are increasingly affected by frictional effects. On some barrier reefs, friction has been shown to be the dominant dissipative process[31]. Wave setup depends strongly on the profile geometry and reef architecture. To define the bottom friction, we build from specific work by Sheppard and others [32] and Nunes and Pawlak[33]on coral reefs and wave damping. Shepard and others[32] have identified friction factors (f_w) based on live coral cover as summarized in Table S3. We have used these recommendations to identify different friction factors by oceanic region (Supplementary Figure 4) based on overall coral condition[34]. We assume the greatest live coral reef cover in the Pacific Islands and Micronesia (f_w=0.2); lower live coral reef cover in the Indian and west Pacific Oceans (f_w=0.16) and lowest overall live coral reef cover in the Caribbean and Latin America (f_w=0.14).

Nearshore hydrodynamics total water level model. The total water level (i.e., flood height) along shorelines is a function of mean sea level, astronomical tide, storm surge, and the run-up of waves[35].The run-up represents the wave-induced motion of the water's edge across the shoreline and is built of two contributions, namely the wave setup at the shoreline and the swash representing oscillations about the setup. The run-up calculation requires obtaining the local wave conditions at the shoreline using the reef wave model above.

Nearshore hydrodynamics computation of wave setup. Wave set-up is a significant contributor to water levels, particularly in coral reefs environments, and hence is an important factor in determining flooding. The wave-setup is obtained from the conservation of mass and the momentum equations [36]. In our one-dimensional setting, the computation of the wave-induce setup is based on the vertically integrated momentum balance equation[37]. Similar

implementations have been used in previous work to evaluate the effect of vegetation on wave-induced setup [7] and in coral reef environments[38]. Comparison of natural forms of reef profiles and idealized horizontal reefs show that the relative setup on natural reef profiles is found to be less than the idealized ones[39,40], partly because they do not consider longshore effects.

Nearshore hydrodynamics computation of wave runup. The 2% exceedance level of wave runup maxima generated by random wave fields on open coast sandy beaches was estimated in Stockdon et al[41] as:

$$R_{u\_Stockdon} = 1.1 \cdot \left(0.35m\sqrt{H_0 L_0} + \frac{\sqrt{0.004H_0L_0+0.563H_0L_0m^2}}{2}\right) \qquad (5)$$

where H_0 is the offshore significant wave height, L_0 represents the deep-water wave length, T_p the peak period, and m the bathymetry slope in the foreshore beach slope. This equation expresses runup as a function of empirical estimates of incident wave setup at the shoreline (first term of the equation) and the swash incident and infragravity band frequency band components (second term of the equation).

The wave setup is calculated from the propagation of waves in the reef model, as described above. For the swash and infragravity band frequency components, we apply the second term of the equation to find their contribution to the total water level as follows:

- Using the wave propagation model over the reef, we calculate the breaking point position (x_b), breaking depth (h_b) and breaking height (H_b). The breaking point depends on the incident wave conditions, the sea level, the reef geometry and friction and is calculated from D_b (equation 2).
- Following Stockdon et al. recommendations we deshoal the wave height to deep water to obtain H_0 L_0 is calculated for the corresponding peak period (T_p).
- The foreshore slope m is obtained for each of the different profiles from the DIVA-GIS dataset (http://www.diva-gis.org/).

In our approach, we assume that Stockdon et al.[41] can be applied to coral reefs as the model was developed to include barred beaches, which resemble coral reef protected beaches. Modifications of the same formula have been applied previously to estimate the effect of vegetated ecosystems on runup[7]. In reef environments, wave runup depends strongly on the reef complexity and architecture[42,43]. An alternative to this approach to calculate the runup swash component would be the application of nonlinear process based models to coral reef environments[43-45]. However these nonlinear models are computationally demanding and require high resolution bathymetry. Therefore, they are only feasible for small spatial domains and a very limited set of sea states. For validation purposes, we have compared the runup predicted by our reef model versus XBeach[42] for an idealized fringing reef profile. XBeach is a state-of-art hydrodynamic model that has been applied in reef environments (see section below **Validation of flooding model**).

Extreme water levels and flood height reconstruction. From the propagations of waves and the calculation of total water levels onshore, the reconstruction of the flood height time series at the most onshore points is based on multi-dimensional interpolation techniques[13]. We apply a Peak Over Threshold method to select extreme flood heights and fit a General Extreme Value Distribution[15] to obtain the flood heights associated with the 10-, 25-, 50- and 100-year return periods. The methodology has been tested in case studies [46,47] and validated with observations showing a good reproduction of time series structure and statistical parameters in shallow water. The methodology is not location dependent and is applicable globally.

Estimating reef benefits. To examine the current value of reefs for coastal protection, we compared flooding under current conditions, “with reef”, to the flooding in a scenario “without reefs”. In our “without reefs” scenario, we do not assume the loss of the entire reef habitat; we

assume only the loss of the top 1m in height across the reef bathymetric profile. Many ecosystem service assessments assume the entire loss of a habitat for estimating benefits. For example, the replacement cost method, which is the most commonly used method for estimating the benefits from mangrove and reef habitats[48], identifies the flood reduction benefits from habitats by estimating the cost of replacing them with seawalls or breakwaters. Many problems have been identified with the replacement method and it provides estimates of values 10 times higher than the recommended Expected Damage Function approach that we follow[48,49].

The "without reefs' scenario is not meant to be a prediction of site-specific trajectories for reefs, but nonetheless this level of loss is already observed to be happening[6,50,51] and is conservative relative to future predictions of reef loss[52-54]. In addition to the widely observed declines in coral cover, growth and condition, all of which affect reef height[55,56], new measures of seafloor elevation show that bioerosion and carbonate dissolution are degrading height across all reef habitats including on reef flats[51]. Damage from storm events can also create losses in reef height of 1-3 m[57,58] and can devastate whole shallow reef frameworks[59]. Past storms have removed many branching and massive corals at the shallowest depths[57,60]. Shallow corals have evolved with intermittent storms and can recover from them, but this is more difficult when reefs are exposed to multiple stressors[24].

Calculating people and assets flooded. We assessed flood heights along each coastal profile and then identified the area flooded within each coastal study unit. We extended the flood heights inland by ensuring hydraulic connectivity between points at a 90m resolution; a significant advance over more common bathtub approaches in earlier global flooding models. From the flooding levels and flooding extent, we calculated the total area of land affected and damages at each study unit. Flooding maps were also intersected with population data[17] after resampling from the original 1 km resolution to the 90 m of the digital elevation model. Existing artificial defenses such as seawalls were not assessed, because data on defenses only exist for a very few areas globally; these built defenses are also less common in tropical, developing nations.

We expanded on earlier approaches to infer built capital from population data[1,18] by identifying the ratio between built capital per capita and the Gross Domestic Product (GDP) per capita for each country[1] in 2011 US$ using information from the World Bank[61]. We filled data gaps for several countries by using the average from countries with similar income levels and affiliation to the Organization for Economic Cooperation and Development (OECD). The overall global mean ratio that we obtained (2.67) is similar to that obtained by Hallegatte and others (2.8)[18]. However, we did identify significant differences in the ratios across some countries and regions (e.g., Cuba - 4.53, Vietnam - 3.22, Australia - 3.17, Philippines - 2.68, United Arab Emirates - 1.98, Micronesia - 1.38).

Assessing damages and estimating annual benefits. We followed existing approaches for assessing the damages to built capital as a function of the flooding level[62]. We calculated the percentage of built capital that has been damaged (D) for a given flooding level h and a certain coefficient k that must be calibrated as $D(h) = h/(h + k)$. This curve indicates that as flooding level increases, the percent of damages to built capital also increases. While there is debate about the right k to choose, we have followed others in using $k = 1.0$, which means that the built capital flooded at 1m of depth loses 50% of its value[62]. We follow standard terminology where the total built capital flooded is the exposure of assets and the value lost is the damages. The economic benefits of flood protection are the avoided damages.

In addition to assessing risk and damages for particular events (e.g., 100-year storm event), we also examined average annual expected loss[63]. To estimate annual risk, we integrated the values under the curve that compares built capital damaged by storm return period, i.e., the integration of the expected damage by the probability of the storm events[62].

Sea level rise. We used regional sea level rise projections and associated uncertainty estimates produced Slangen, et al. [19] on a 1^ox1^o grid. Projections account for changing ocean circulation, increased heat uptake and atmospheric pressure in 21 CMIP5 climate models[64], combined with model- and observation-based regional contributions of land ice, groundwater depletion and glacial isostatic adjustment, including gravitational effects due to mass redistribution. In our analysis we select the estimates for the Representative Concentration Pathway 8.5, which corresponds to a global mean surface temperature of 2.7-5.4^o C and a global mean sea level rise of 0.71 ± 0.28 m (from 1986-2005 to 2081-2100). Regional variations deviate up to 20% higher from the global mean in the subtropical and equatorial regions.

We calculate the regional sea level rise at the end of the century, at each profile, by storm return period and add these values to the estimates of the total water level offshore. The wave propagations over the reefs are recalculated and the flood height statistics reconstructed as described above.

Sensitivity analyses. Hinkel, et al. [62] carried out a global analysis of coastal flood damage under sea level rise. Our two approaches are in general very similar with some differences in the global data sets selected and the fact that we use a process-based flooding model. They analyzed the sensitivity of the flooding models to bathymetry and topography and found that corrections needed to reduce uncertainties are feasible at local scales but cannot currently be applied at global scale for technical constraints. This conclusion is applicable to our analysis.

To identify the sensitivity of our flood height estimates to the factors affecting wave run-up, we examined two major reef types: barrier and fringing reefs. From our global set, we selected 1000 profiles for each reef type that were representative of their geographic variations (2000 profiles total). We examined the effects of reef friction (C_{f_reef}), wave breaking (γ_{coral}), and water depth (h_{coral}) on wave run-up across the 2000 profiles. We varied the reef friction parameter in increasing steps of 0.01, from 0.08-0.20, as taken from Sheppard and others[32], which cover the range of friction factors from sand to 75%-100% live coral. We varied the wave breaking parameter proposed by Yao et al. (2013) for $\gamma_{coral} = 0.2$ to 1.2, in increasing steps of 0.1. We varied water depth above the reef in increasing steps of 0.1 m, from 0.1 m to 1 m.

Supplementary Table 4 summarizes the results of the sensitivity analysis. For each of the reef types, we show the maximum, minimum and mean run-up variation observed across all the incremental changes in the parameters (e.g., for the water depth analysis, the table, first column, summarizes the results from 1,000 profiles at each of 10 water depths for a total of 10,000 runs for the barrier reef type).

For the reef friction factor, the results indicate that the overall effects of changes in friction on run-up are very low, with a 13% difference across the entire range of factors examined (a ~1% change in run-up for each increment in the friction factor that we tested).

For the wave breaking coefficient, results show that run-up varies on average from 1.71%-2.53% per increment. Based on the formulation by Yao et al. (2013), and considering a variation in the breaking coefficient from 0.2 and 0.6, our results indicate a total variation in results from 6% to 10% (Supplementary Table 4).

Changes in water depth resulted in slightly larger variations in run up in our sensitivity analysis. On average, and depending on the type of reef, every 10 cm increase in water depth results in an increase of 1.68%-2.75% in run-up. This is mainly due to the effect that depth has on breaking or shoaling conditions, and clearly indicates the importance of an accurate bathymetry (Supplementary Table 4).

In sum for the sensitivity analysis, we conclude that the range of friction coefficients recommended by Sheppard and others[32] produces a variation of 13% in wave run-up, whereas

variations in the wave breaking coefficient recommended by Yao et al. (2013) introduces variations up to 10%. Changes in water depth may change run-up from 16% to 27% over a 1 m range. Hence it can be concluded that the most relevant aspect to reduce uncertainties in the results is using high quality bathymetry with a good definition of coral reef height. The importance of bathymetry is relevant for all coastal flooding models.

Validation of the wave and flooding model. Our physical modeling approach relies on linear wave theory to calculate the effect of reefs on waves and wave setup. We use a modification of an empirical formula to estimate the swash component of run-up (see above) that was originally calculated for beach environments. Models based on the same equation (e.g., SWAN) have been used to study the effect of reefs on flooding at regional scales[28,65,66]. More complex but still 1D models have been used for the study of the hydrodynamics of coral reefs in more detail at specific sites and they also use transect-based approaches[42].

We compare and validate our modeling approach against a more complex model for reef environments, XBeach[43,67] in a theoretical fringing reef profile. The comparison was performed in a frictionless setting to focus on comparisons of wave breaking and run-up. XBeach was originally derived from sandy beaches and has hitherto been successfully applied to predict erosion and overwash under hurricane forcing[68] and more recently, successfully applied in reef environments[42,43].

We considered three different reef depths or sea levels (0, 1.5 and 3m) and three different offshore significant wave heights (1, 3 and 6m) and estimated flood height at the shore with each model. The comparison shows that the flood height values of our approach are comparable to those from a higher resolution model (Supplementary Figure 7, r^2=0.978) given the simple geometries that are characteristic of the global bathymetric data.

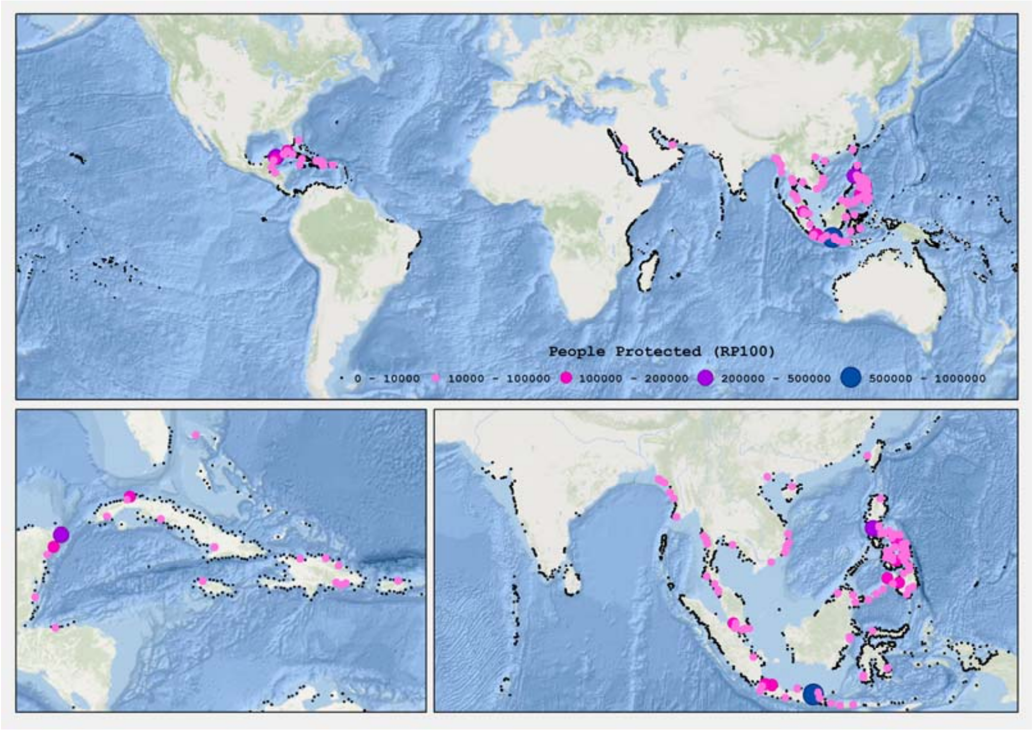

Supplementary Figure 1. The value of coral reefs for flood protection to people. Circles represent the additional people flooded in a100-yr event without (the top 1m of) coral reefs. Black and colored dots represent the 20-km coastal analysis units.

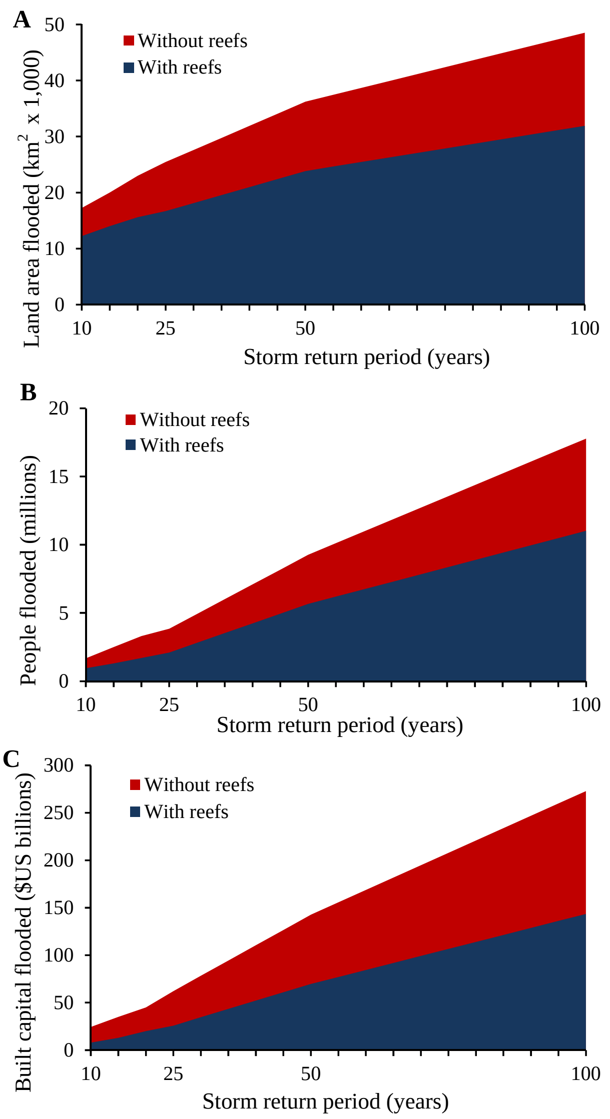

Supplementary Figure 2 | Global coastal protection from coral reefs. The values are the expected people, area and built capital flooded with and without reefs by storm event return period globally. The difference between the curves represents the avoided impacts. In all graphs, lower lines are predicted impact given current reef profiles and upper lines are impacts with 1m decreases in the bathymetric profile of reefs and associated loss of friction.

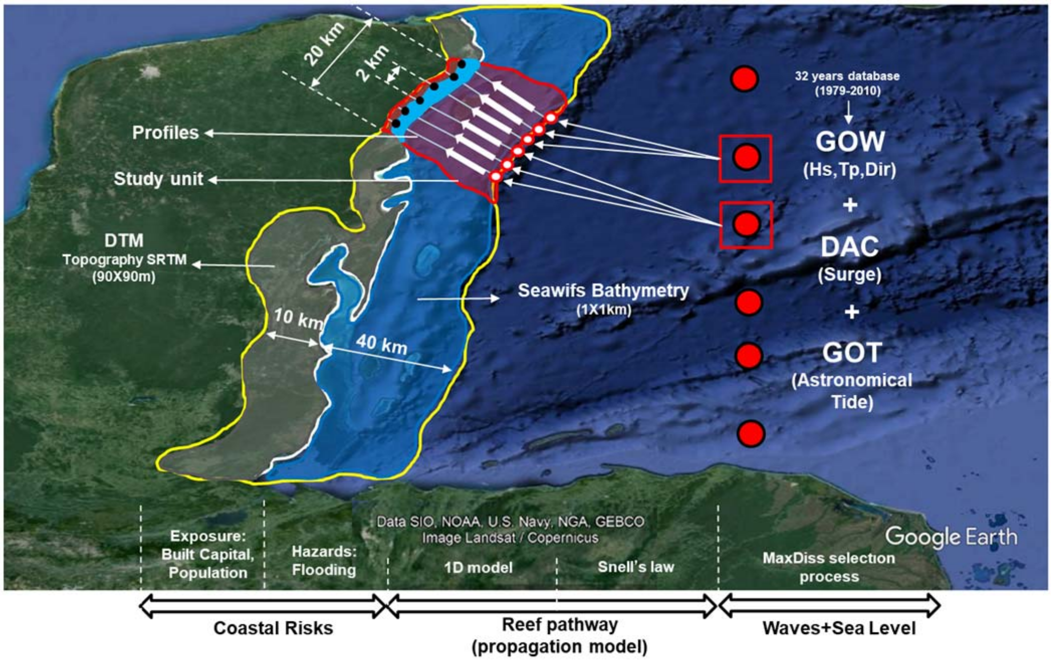

Supplementary Figure 3 | Key steps and data for estimating the flood protection provided by reefs. Data on waves, tides, surges and mean sea levels are combined to assess the total water levels offshore. Nearshore hydrodynamics are obtained from propagation of offshore conditions along 2 km coastal profiles. Flood heights are calculated at the shoreline and extended inland to define flooding envelopes for present and reef loss situations across the 20-km study units. Then the land, people and built capital within the flooding maps are identified. GOW is Global Ocean Waves, DAC is Dynamic Atmospheric Correction, and GOT is Global Ocean Tides. Map Data © 2018 Google.

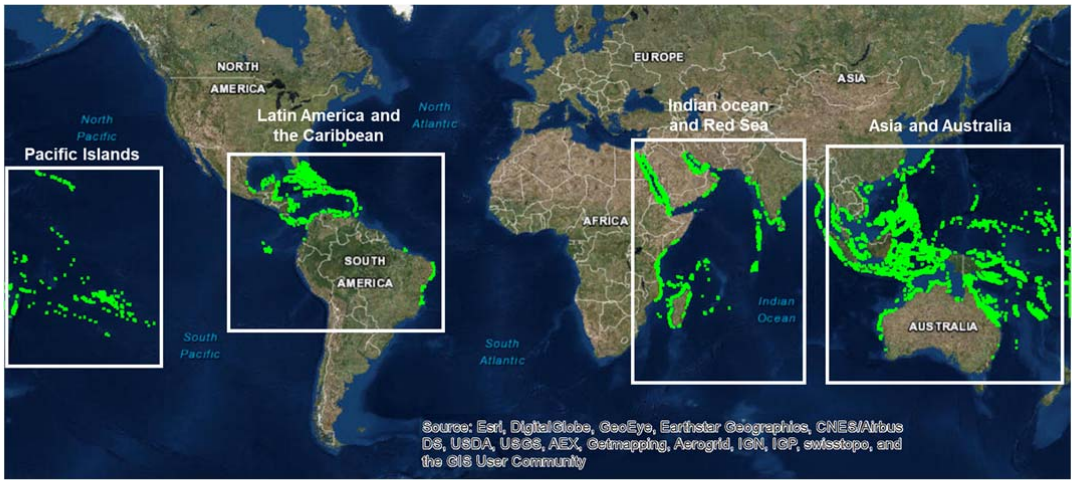

Supplementary Figure 4 | Coral reefs and analysis regions. Green dots correspond to coral reef locations globally. White frames show the 4 coral reef regions. Map Data © 2018 Google.

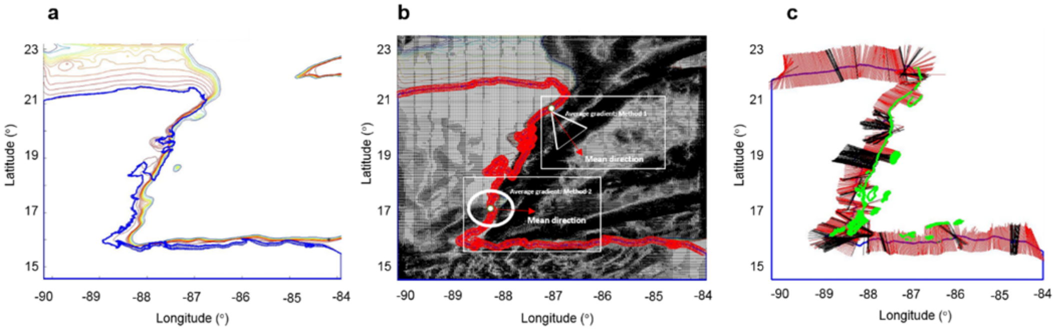

Supplementary Figure 5 | Methods to generate profiles. Panels outline the workflow to obtain coastal profiles for the analysis: (a) bathymetry and coastline preprocessing and smoothing – profiles are aligned with the bathymetry gradients; (b) selection of offshore hydrodynamic points from global waves and sea level datasets; and (c) exclusion of profiles that overlap one another (black lines).

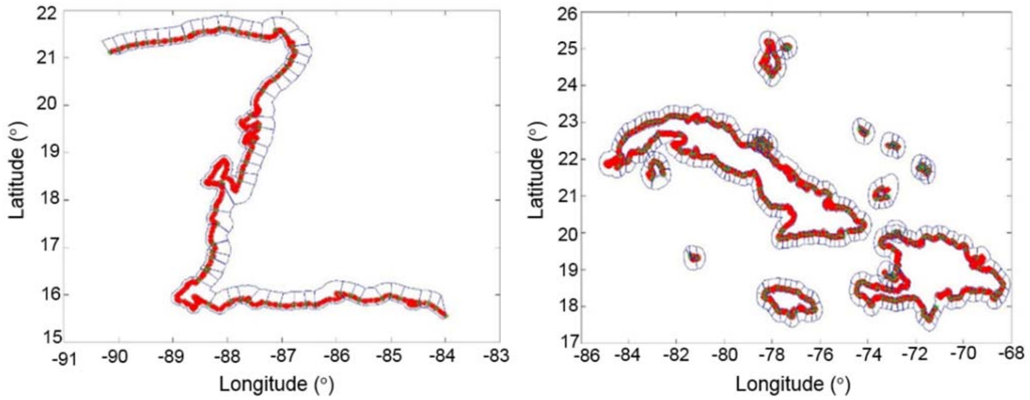

Supplementary Figure 6 | Examples of study units. Study Units in Mesoamerica (left panel) and Cuba and Hispaniola Islands (right panel). The blue polygons identify the study units. The width of the study units is approximately 20 km of coastline, although it depends on coastal sinuosity, except for small islands where accurate definition of the shoreline required more refined study units.

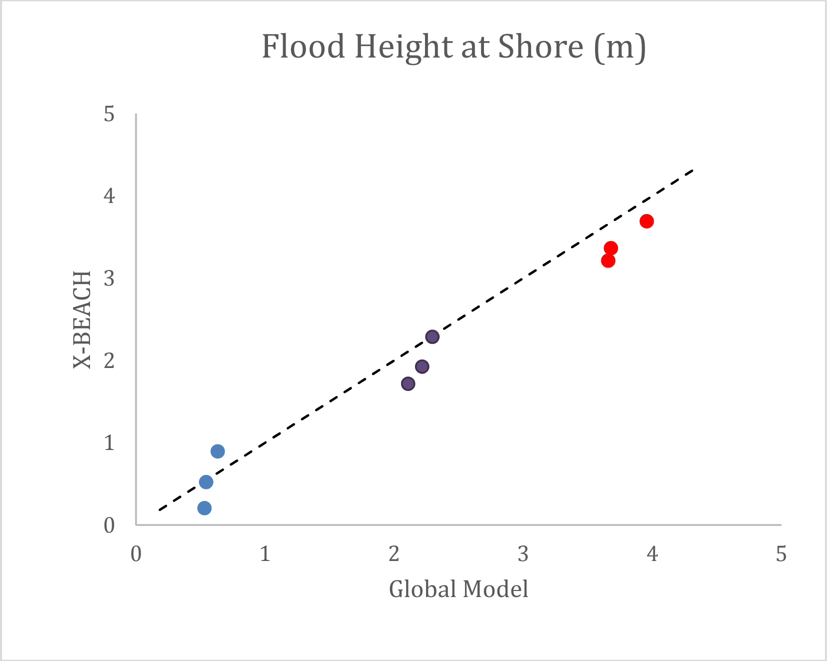

Supplementary Figure 7: Comparison of flood height at the shore predicted by XBeach and the global model in this paper. We examined results for a fringing reef profile under 3 different reef depths (0, -1.5m, - 3 meters) represented by the different colors and three different significant wave heights (H_S= 1, 3 and 6 meters).

Supplementary Table 1. The difference in built capital flooded with and without reefs for 100-year events. The values are the averted flooding of built capital in absolute terms ($US billions) and relative to total national built capital for each country.

Built capital flooded (100-yr)			% of the national built capital		
1.	Indonesia	36.48	1.	Cayman Islands	6.81
2.	Philippines	31.14	2.	Belize	4.28
3.	Malaysia	27.07	3.	Grenada	3.76
4.	Cuba	19.04	4.	Bahamas	3.70
5.	Mexico	18.87	5.	Jamaica	2.67
6.	United Arab Emirates	7.84	6.	Cuba	2.61
7.	Saudi Arabia	7.29	7.	Philippines	2.37
8.	United States	6.55	8.	Dom. Republic	2.19
9.	Dom. Republic	4.43	9.	Malaysia	1.54
10.	Thailand	2.86	10.	Antigua & Barbuda	1.50
11.	Jamaica	2.43	11.	Seychelles	1.14
12.	Vietnam	2.26	12.	Turks and Caicos	0.88
13.	Taiwan	1.83	13.	New Caledonia	0.83
14.	Myanmar	1.04	14.	Pitcairn Islands	0.74
15.	Bahamas	0.77	15.	Indonesia	0.72

Supplementary Table 2 | Ranking of countries that receive the most flood protection benefits from reefs in terms of avoided flooding of land and people annually. The table ranks countries by the absolute and percent differences in land area and people that would be flooded with and without reefs. See Table 1 for differences in built capital flooded.

	ANNUAL EXPECTED AREA FLOODED (km^2)				ANNUAL EXPECTED PEOPLE FLOODED			
	Absolute		% of the total in country		Absolute		% of the total in country	
1	Philippines	88.45	Cayman Islands	0.968	Philippines	73162	Cayman Islands	0.2402
2	Indonesia	83.20	Turks & Caicos	0.609	Indonesia	56034	Belize	0.1973
3	Cuba	79.51	Bahamas	0.224	Mexico	15391	Grenada	0.1318
4	Mexico	38.02	Kiribati	0.219	Malaysia	12340	Bahamas	0.1015
5	Bahamas	31.07	Antigua & Barbuda	0.188	Cuba	8061	Philippines	0.0720
6	Myanmar	26.21	Bermuda	0.085	Myanmar	7166	Cuba	0.0705
7	Dom. Republic	18.41	Cuba	0.072	Dom.Republic	6872	Dom. Republic	0.0645
8	Saudi Arabia	16.89	British Indian Ocean Territory	0.057	Vietnam	6319	Jamaica	0.0615
9	Malaysia	12.70	Maldives	0.053	Thailand	2077	Antigua & Barbuda	0.0512
10	Thailand	8.99	Dom. Republic	0.038	Saudi Arabia	1865	Malaysia	0.0399
11	Vietnam	8.49	Philippines	0.030	Jamaica	1687	Turks & Caicos	0.0249
12	Papua New Guinea	5.82	U.S. VI	0.028	Haiti	1323	Indonesia	0.0220
13	Honduras	4.83	Grenada	0.019	United States	1102	Solomon Islands	0.0177
14	Belize	4.27	Belize	0.019	Honduras	766	Guadeloupe	0.0174
15	United States	3.88	Guadeloupe	0.017	Belize	663	Myanmar	0.0133
16	Jamaica	2.56	Tonga	0.014	Taiwan	595	Haiti	0.0125
17	Turks & Caicos	2.54	Martinique	0.014	Bahamas	392	Mexico	0.0123
18	Cayman Islands	2.51	Jamaica	0.013	Papua New Guinea	344	Maldives	0.0111
19	Sudan	2.22	Marshall Islands	0.010	Iran	243	Bermuda	0.0099
20	Madagascar	1.92	Guam	0.007	Madagascar	222	Martinique	0.0096
21	Oman	1.87	Saint Lucia	0.007	India	214	Honduras	0.0093
22	Kiribati	1.78	N. Mariana Is.	0.005	Somalia	172	Pitcairn Islands	0.0092
23	Taiwan	0.94	Seychelles	0.005	Cayman Islands	147	Vietnam	0.0069
24	Egypt	0.87	Indonesia	0.004	Grenada	137	Saint Lucia	0.0065
25	Antigua & Barbuda	0.83	Honduras	0.004	Solomon Islands	104	Saudi Arabia	0.0062
26	Australia	0.70	Myanmar	0.004	Egypt	99	Papua New Guinea	0.0046
27	India	0.67	Malaysia	0.004	Venezuela	93	China	0.0044
28	Fiji	0.63	Fiji	0.003	Cambodia	86	U.S.V.I.	0.0041
29	Eritrea	0.51	Taiwan	0.003	Guadeloupe	71	Tonga	0.0040
30	Solomon Island	0.50	Vietnam	0.003	Sri Lanka	50	Thailand	0.0030

Supplementary Table 3 | The relationship between reef condition and friction factors as proposed by Sheppard and others[6]. This relationship informed the use of friction factors in the regional analyses**.**

Coral reef condition	% of coral reef condition	Friction factor (f_w)
Dead coral (Sandy bottom)	100	0.08
Smooth rock or coral pavement	75-100	0.1
Seagrass or algal turf	75-100	0.1
Smooth rock or coral pavement + coral rubble	50-100	0.12
Live coral or Dead uneroded coral or Tall boulders >30cm	10-25	0.14
Live coral or Dead uneroded coral or Tall boulders>30cm	25-50	0.16
Live coral or Dead uneroded coral or Tall boulders>30cm	50-75	0.18
Live coral or Dead uneroded coral or Tall boulders>30cm	75-100	0.2

Supplementary Table 4 | Results of Sensitivity Analyses

	% change in run-up per increment	
	Barrier reef	Fringing reef
Reef Friction<br>ΔC_{f_reef} =0.01	$avg = 1.09\%$<br>$max = 3.04\%$<br>$min = 0.10\%$	$avg = 0.85\%$<br>$max = 1.82\%$<br>$min = 0.15\%$
Wave Breaking<br>$\Delta \gamma_{coral}$ =0.1	$avg = 1.71\%$<br>$max = 5.08\%$<br>$min = 0.10\%$	$avg = 2.53\%$<br>$max = 6.61\%$<br>$min = 0.33\%$
Water Depth<br>Δh_{coral} =0.1m	$avg = 2.75\%$<br>$max = 9.97\%$<br>$min = 0.11\%$	$avg = 1.68\%$<br>$max = 4.13\%$<br>$min = 0.10\%$

Supplementary References:

1 Reguero, B. G., Losada, I. J., Diaz-Simal, P., Mendez, F. J. & Beck, M. W. Effects of climate change on exposure to coastal flooding in Latin America and the Caribbean. *Plos One* **10** (2015).

2 Losada, I. J. *et al.* Long-term changes in sea-level components in Latin America and the Caribbean. *Global and Planetary Change* **104**, 34-50 (2013).

3 Reguero, B. G., Menéndez, M., Mendez, F. J., Minguez, R. & Losada, I. J. A Global Ocean Wave (GOW) calibrated reanalysis from 1948 onwards. *Coastal Engineering* **65**, 38-55 (2012).

4 Reguero, B. G., Mendez, F. J. & Losada, I. J. Variability of multivariate wave climate in Latin America and the Caribbean. *Global and Planetary Change* **100**, 70-84 (2013).

5 Cid, A., Castanedo, S., Abascal, A. J., Menéndez, M. & Medina, R. A high resolution hindcast of the meteorological sea level component for Southern Europe: the GOS dataset. *Climate Dynamics* **43**, 1-18 (2014).

6 Sheppard, C., Dixon, D. J., Gourlay, M., Sheppard, A. & Payet, R. Coral mortality increases wave energy reaching shores protected by reef flats in the Seychelles. *Estuarine Coastal and Shelf Science* **64**, 223-234 (2005).

7 Guannel, G. *et al.* Integrated modeling framework to quantify the coastal protection services supplied by vegetation. *Journal of Geophysical Research-Oceans* **120**, 324-345 (2015).

8 Dean, R. G. & Dalrymple, R. A. *Water wave mechanics for engineers and scientists*. (Prentice-Hall, 1991).

9 Monismith, S. G., Herdman, L. M. M., Ahmerkamp, S. & Hench, J. L. Wave transformation and wave-driven flow across a steep coral reef. *J Phys Oceanogr* **43**, 1356-1379 (2013).

10 Monismith, S. G. Hydrodynamics of coral reefs. *Annu Rev Fluid Mech* **39**, 37-55 (2007).

11 Stumpf, R. P., Arnone, R. A., Gould, R. W. J., Martinolich, P. & Ransibrahmanakul, V. in *Algorithm updates for the fourth SeaWiFS data reprocessing, NASA Tech. Memo. 206892 Vol. 22* (eds S.B. Hooker & E.R. Firestone) (NASA Goddard Space Flight Center, 2003).

12 Stumpf, R. H. *et al.* SeaWiFS spies reefs. *Reef Encounter* **26**, 29-30 (1999).

13 Camus, P., Mendez, F. J. & Medina, R. A hybrid efficient method to downscale wave climate to coastal areas. *Coastal Engineering* **58**, 851-862 (2011).

14 Menéndez, M. & Woodworth, P. L. Changes in extreme high water levels based on a quasi-global tide-gauge data set. *Journal of Geophysical Research: Oceans* **115**, 1-15 (2010).

15 Mendez, F. J., Menéndez, M., Luceno, A. & Losada, I. J. Estimation of the long-term variability of extreme significant wave height using a time-dependent Peak Over Threshold (POT) model. *Journal of Geophysical Research-Oceans* **111**, 2156-2202 (2006).

16 Rabus, B., Eineder, M., Roth, A. & Bamler, R. The shuttle radar topography mission—a new class of digital elevation models acquired by spaceborne radar. *ISPRS Journal of Photogrammetry and Remote Sensing* **57**, 241-262 (2003).

17 Bright, E. A., Coleman, P. R., Rose, A. N. & Urban, M. L. LandScan 2011. *Digital dataset, Oakridge National Laboratory, TN, USA, web. ornl. gov/sci/landscan/index. shtml* (2012).

18 Hallegatte, S., Green, C., Nicholls, R. J. & Corfee-Morlot, J. Future flood losses in major coastal cities. *Nature Climate Change* **3**, 802-806 (2013).

19 Slangen, A. B. A. *et al.* Projecting twenty-first century regional sea-level changes. *Climatic Change* **124**, 317-332 (2014).

20 Camus, P., Mendez, F. J., Medina, R. & Cofiño, A. S. Analysis of clustering and selection algorithms for the study of multivariate wave climate. *Coastal Engineering* **58**, 453-462 (2011).
21 Reguero, B. G., Menéndez, M., Méndez, F. J., Mínguez, R. & Losada, I. J. A Global Ocean Wave (GOW) calibrated reanalysis from 1948 onwards. *Coastal Engineering* **65**, 38-55 (2012).
22 Church, J. A., White, N. J., Coleman, R., Lambeck, K. & Mitrovica, J. X. Estimates of the regional distribution of sea level rise over the 1950-2000 period. *Journal of Climate* **17**, 2609-2625 (2004).
23 Church, J. A. & White, N. J. Sea-level rise from the late 19th to the early 21st century. *Surveys in Geophysics* **32**, 585-602 (2011).
24 Cid Carrera, A. Modelado Numérico y Estadístico de la Componente Meteorológica del Nivel del Mar a Escala Regional y Global. Numerical and Statistical Modelling of the Atmospheric Surge at Regional and Global Scale. (2015).
25 Carrère, L. & Lyard, F. Modeling the barotropic response of the global ocean to atmospheric wind and pressure forcing - comparisons with observations. *Geophysical Research Letters* **30**, DOI: 10.1029/2002GL016473 (2003).
26 Amante, C. & Eakins, B. W. *ETOPO1 1 arc-minute global relief model: procedures, data sources and analysis*. (National Oceanic and Atmospheric Administration, 2009).
27 *SWAN User Manual*. (Delft University of Technology, 2009).
28 Guannel, G., Arkema, K., Ruggiero, P. & Verutes, G. The power of three: coral reefs, seagrasses and mangroves protect coastal regions and increase their resilience. *PLoS ONE* **11(7)**, e0158094 (2016).
29 Thornton, E. B. & Guza, R. T. Transformation of wave height distribution. *Journal Geophysical Research* **88**, 5925-5938 (1983).
30 Yao, Y., Huang, Z., Monismith, S. & Lo, E. Y. Characteristics of monochromatic waves breaking over fringing reefs. Journal of Coastal Research. **29**, 94-104 (2013).
31 Lowe, R. J. *et al.* Spectral wave dissipation over a barrier reef. *Journal of Geophysical Research-Oceans* **110**, 4001-4001 (2005).
32 Sheppard, C., Dixon, D. J., Gourlay, M., Sheppard, A. & Payet, R. Coral mortality increases wave energy reaching shores protected by reef flats: Examples from the Seychelles. *Estuarine, Coastal and Shelf Science* **64**, 223-234 (2005).
33 Nunes, V. & Pawlak, G. Observations of bed roughness of a coral reef. *Journal of Coastal Research* **24**, 39-50 (2008).
34 Burke, L., Reytar, K., Spalding, M. & Perry, A. *Reefs at risk revisited*. (WRI, 2011).
35 Losada, I. J. *et al.* Long-term changes in sea-level components in Latin America and the Caribbean. *Global and Planetary Change* **104**, 34-50 (2013).
36 Longuet-Higgins, M. S. & Stewart, R. W. The changes in amplitude of short gravity waves on steady non-uniform currents. *Journal of Fluid Mechanics* **10**, 529-549 (1961).
37 Holthuijsen, L. H. *Waves in oceanic and coastal waters*. (Cambridge University Press, 2007).
38 Massel, Sr. & Gourlay, M. R. On the modelling of wave breaking and set-up on coral reefs. *Coastal Engineering* **39**, 1-27 (2000).
39 Gourlay, M. R. Wave set-up on coral reefs. 2. set-up on reefs with various profiles. *Coastal Engineering* **28**, 17-55 (1996).
40 Gourlay, M. R. Wave set-up on coral reefs. 1. Set-up and wave-generated flow on an idealised two dimensional horizontal reef. *Coastal Engineering* **27**, 161-193 (1996).
41 Stockdon, H., Holman, R. A., Howd, P. & Sallenger, A. Empirical parameterization of setup, swash, and runup. *Coastal Engineering* **53**, 573-588 (2006).
42 Van Dongeren, A. *et al.* Numerical modeling of low-frequency wave dynamics over a fringing coral reef. *Coastal Engineering* **73**, 178-190 (2013).
43 Quataert, E., Storlazzi, C., van Rooijen, A., Cheriton, O. & van Dongeren, A. The influence of coral reefs and climate change on wave-driven flooding of tropical coastlines. *Geophysical Research Letters* **42**, 6407-6415 (2015).

44 Monismith, S. G., Rogers, J. S., Koweek, D. & Dunbar, R. B. Frictional wave dissipation on a remarkably rough reef. *Geophysical Research Letters* **42**, 4063-4071 (2015).

45 Buckley, M. L., Lowe, R. J., Hansen, J. E. & Van Dongeren, R. Wave Setup over a Fringing Reef with Large Bottom Roughness. *J Phys Oceanogr* **46**, 2317-2333 (2016).

46 Guanche, Y., Camus, P., Guanche, R., Mendez, F. J. & Medina, R. A simplified method to downscale wave dynamics on vertical breakwaters. **71**, 68-77 (2013).

47 Camus, P., Mendez, F. J., Medina, R., Tomas, A. & Izaguirre, C. High resolution downscaled ocean waves (DOW) reanalysis in coastal areas. *Coastal Engineering* **72**, 56-68 (2013).

48 World Bank. Managing coasts with natural solutions: Guidelines for measuring and valuing the coastal protection services of mangroves and coral reefs. (eds M. W. Beck, G-M Lange). (World Bank, Washington, DC, 2016).

49 Barbier, E. B. Valuing the storm protection service of estuarine and coastal ecosystems. *Ecosystem Services* **11**, 32-38 (2015).

50 Alvarez-Filip, L., Dulvy, N. K., Gill, J. A., Cote, I. M. & Watkinson, A. R. Flattening of Caribbean coral reefs: region-wide declines in architectural complexity. *P R Soc B* **276**, 3019-3025 (2009).

51 Yates, K. K., Zawada, D. G., Smiley, N. A. & Tiling-Range, G. Divergence of seafloor elevation and sea level rise in coral reef regions. *Biogeosciences* **14**, 1739-1772 (2017)..

52 Wong, P. P. *et al.* in *Climate change 2014: Impacts, adaptation, and vulnerability : Working Group II contribution to the fifth assessment report of the IPCC* (eds C. B. Field, V. R. Barros, & IPCC Working Group II) 361-409 (Cambridge University Press, 2014).

53 *Turn down the heat : confronting the new climate normal*. (World Bank, 2014).

54 Hoegh-Guldberg, O. *et al.* Coral reefs under rapid climate change and ocean acidification. *Science* **318**, 1737-1742 (2007).

55 Graham, N. A. J., Jennings, S., MacNeil, M. A., Mouillot, D. & Wilson, S. K. Predicting climate-driven regime shifts versus rebound potential in coral reefs. *Nature* **518**, 94-+ (2015).

56 Bruno, J. F. & Selig, E. R. Regional Decline of Coral Cover in the Indo-Pacific: Timing, Extent, and Subregional Comparisons. *Plos One* **2** (2007).

57 Harmelin-Vivien, M. L. The effects of storms and cyclones on coral reefs: a review. *Journal of Coastal Research* **12**, 211-231 (1994).

58 Woodley, J. D. *et al.* Hurricane Allen's impact on Jamaican coral reefs. *Science* **214**, 749-755 (1981).

59 Fabricius, K. E. *et al.* Disturbance gradients on inshore and offshore coral reefs caused by a severe tropical cyclone. *Limnol Oceanogr* **53**, 690-704 (2008).

60 Puotinen, M., Maynard, J. A., Beeden, R., Radford, B. & Williams, G. J. A robust operational model for predicting where tropical cyclone waves damage coral reefs. *Sci Rep-Uk* **6**, 26009-26009 (2016).

61 World Bank. *The changing wealth of nations: measuring sustainable development in the new millennium*. (World Bank, 2011).

62 Hinkel, J. *et al.* Coastal flood damage and adaptation costs under 21st century sea-level rise. *P Natl Acad Sci USA* **111**, 3292-3297 (2014).

63 Hallegatte, S. *et al.* Assessing climate change impacts, sea level rise and storm surge risk in port cities: a case study on Copenhagen. *Climatic Change* **104**, 113-137 (2011).

64 Taylor, K., Stouffer, R. & Meehl, G. An overview of CMIP5 and the experiment design. *Bulletin of the American Meteorological Society* **93**, 485-498 (2012).

65 de Alegria-Arzaburu, A. R., Marino-Tapia, I., Enriquez, C., Silva, R. & Gonzalez-Leija, M. The role of fringing coral reefs on beach morphodynamics. *Geomorphology* **198**, 69-83 (2013).

66 Arkema, K. K. *et al.* Embedding ecosystem services in coastal planning leads to better outcomes for people and nature. *P Natl Acad Sci USA* **112**, 7390-7395 (2015).

67 Roelvink, D. *et al.* Modelling storm impacts on beaches, dunes and barrier islands. *Coastal Engineering* **56**, 1133-1152 (2009).
68 McCall, R. T. *et al.* Two-dimensional time dependent hurricane overwash and erosion modeling at Santa Rosa Island. *Coastal Engineering* **57**, 668-683 (2010).
