# Supplementary material for: The global flood protection savings provided by coral reefs
Source: Nat Commun. 2018 Jun 12;9:2186. doi: 10.1038/s41467-018-04568-z (PMC5997709; doi:10.1038/s41467-018-04568-z)
Supplement: Supplementary file 3 — Description of Additional Supplementary Files [file 41467_2018_4568_MOESM3_ESM.pdf]

Description of Additional Supplementary Files

File Name: Supplementary Data 1
Description: A summary of the models, equations, and assumptions in the global analyses of the flood reduction value of coral reefs (Beck et al. 2018) with a comparison of their benefits and limitations relative to approaches that are feasible in local or smaller scale studies.
